# Whole-genome analysis of *escherichia coli* isolated from captive giant pandas (*ailuropoda melanoleuca*) at the Dujiangyan base of the China conservation and research center for the giant panda, Sichuan, China

**DOI:** 10.1186/s13028-025-00812-x

**Published:** 2025-05-29

**Authors:** Hongyan Yu, Mengru Zhao, Maolin Lu, Hongjia Li, Shu Fang, Ruisi Zhang, Tianlu Liu, Zhiyou Lü, Mengchao Zhou, Yaxian Lu, Tongzuo Zhang, Zhijun Hou

**Affiliations:** 1 College of Wildlife and Protected Area, Northeast Forestry University, Harbin, People’s Republic of China; 2 Key Laboratory of Adaptation and Evolution of Plateau Biota, Northwest Institute of Plateau Biology, Chinese Academy of Sciences, Xining, China

**Keywords:** *Escherichia coli*, Giant panda, Multiple resistance genes, Virulence factors, Whole-genome sequencing

## Abstract

The giant panda (*Ailuropoda melanoleuca*) is a rare and vulnerable species facing threats from bacterial infections. The extensive use of antibiotics in disease prevention has raised concerns about antibiotic resistance, which reduces treatment efficacy and poses environmental and public health risks. This study aims to analyze the resistance profiles of *Escherichia coli* (*E. coli*) in captive pandas, providing insights into the antibiotic resistance genes within their gut microbiota. Twenty-two *E. coli* isolates were obtained from the feces of 22 captive giant pandas, and whole-genome sequencing was conducted. Sequence types and evolutionary relationships were determined through Multi-Locus Sequence Typing (MLST) and core genome Multi-Locus Sequence Typing (cgMLST), while virulence and resistance genes were identified. Core genome SNP analysis was performed to establish genetic relationships, and AST was conducted to evaluate antibiotic resistance profiles. Whole-genome sequencing revealed 15 distinct sequence types (STs), with ST48 (22.7%, 5 isolates) and ST212 (18.2%, 4 isolates) identified as the dominant lineages. Further resolution using cgMLST revealed that ST48 encompassed multiple cgSTs distributed across different evolutionary branches, while all ST212 isolates belonged to a single cgST. These results demonstrate the utility of cgMLST in resolving genetic diversity and isolate relationships within dominant lineages. Core genome SNP-based phylogenetic analysis grouped isolates with similar serotypes and STs, including a pathogenic cluster closely related to a panda-derived *E. coli* isolate (AMSHJX04). A total of 88 virulence genes (average 52 per isolate) were widespread, including those involved in iron acquisition systems (*yagZ/Y*), fimbriae-associated genes (*fimA/H*), and type II secretion systems (*gspM/K*). Antimicrobial susceptibility testing (AST) showed 59.1% resistance to ciprofloxacin, 18.2% to norfloxacin, and 13.6% to tetracycline, while all isolates remained sensitive to gentamicin and amikacin. Resistance profiling identified 78 resistance genes, primarily efflux pumps (*acrB/D*, *emrA/B*) and tetracycline-related mutations (*emrK/Y*). The gut microbiota of captive giant pandas harbors multiple antibiotic resistance genes, indicating a risk of resistance gene spread. Diverse sequence types, virulence genes, and phylogenetic relationships reveal the genetic complexity of *E. coli* in this species. Some isolates showed genetic similarities to pathogenic *E. coli*, emphasizing the need for continuous bacterial monitoring. This threatens antibiotic efficacy, increases infection risks, and complicates health management, underscoring the urgency of addressing resistance in panda conservation.

**Supplementary Information:**

The online version contains supplementary material available at 10.1186/s13028-025-00812-x.

## Findings

The intestinal microbiota of captive giant pandas (*Ailuropoda melanoleuca*) influences their health, yet bacterial infections pose significant threats [1]. *Klebsiella pneumoniae* and *Campylobacter jejuni* are established enteric pathogens. Virulence gene-positive *E. coli* isolates, in contrast, may act as opportunistic pathogens, causing intestinal inflammation and diarrhea with severe outcomes in compromised hosts [2, 3]. Antibiotic overuse in treating such infections exacerbates multidrug resistance (MDR), with resistance genes spreading via mobile genetic elements [4]. Current MDR studies in giant pandas focus on fecal-derived *E. coli*, revealing resistance rates exceeding those of wild animals (e.g., wild Amur tiger) but remaining lower than in livestock (e.g., commercial chickens) —likely due to dietary specialization and limited antibiotic exposure [5], however, human proximity in captivity may elevate resistance risks [6]. *E. coli* from pandas predominantly carries adhesion-associated virulence genes (e.g., *sfa/foc*, *papC*), critical for pathogenicity [7]. While traditional methods have identified resistance and virulence traits, whole-genome sequencing (WGS) provides unparalleled resolution to simultaneously map resistance genes, virulence factors, and genomic contexts (e.g., plasmids, transposons), revealing transmission and evolutionary insights [8]. Here, we apply WGS to 22 *E. coli* isolates, obtained from fecal samples of 22 captive giant pandas, analyzing MDR profiles and virulence gene features to enhance pathogen surveillance and antibiotic stewardship in conservation.

With authorization from the China Conservation and Research Center for the Giant Panda, Dujiangyan Base, fecal samples were collected from 22 captive giant pandas (housed individually/in small groups), with each sample documented to represent distinct individuals (see Additional file 1). Fecal samples were collected immediately after defecation from enclosure floors using sterile spatulas to avoid substrate contact. The samples were aseptically transferred into cryovials, kept under cooled conditions during transport to the laboratory, and processed for subsequent analyses, thereby ensuring no disruption to the animals’ usual routines. First, fecal samples were streaked onto MacConkey agar (HopeBio, Qingdao, China) and incubated at 37 °C for 12 h. Pink colonies were subcultured on Eosin Methylene Blue (EMB) agar (HopeBio). Metallic-green colonies were purified on Nutrient Agar (Luqiao, Beijing, China). Biochemical identification used Enterobacteriaceae test kits (HB-ENT-20, HopeBio), including indole, citrate, and MR-VP assays. Genomic DNA was extracted with a TIANamp Bacteria DNA Kit (Tiangen, Beijing, China). PCR products of 16 S rRNA genes were sequenced by Comate Bioscience (Changchun, China) and matched to NCBI databases with greater than 99% similarity. Before library preparation, DNA was fragmented and screened, followed by library preparation using the MGIEasy Universal DNA Library Preparation Kit (MGI, Shenzhen, China). The process included end-repair, dA-tailing, ligation, purification, PCR amplification, quality control, denaturation, single-stranded cyclization, enzymatic digestion, and final purification. The products were then sequenced with 150 bp paired-end reads on the MGISEQ-2000 sequencer (BGI, Shenzhen, China).

To ensure the reliability of downstream genomic analyses, raw sequencing reads were first evaluated for quality using FastQC 0.11.9 (https://www.bioinformatics.babraham.ac.uk/projects/fastqc/). Data were filtered with fastp 0.23.2 to remove adapters, primers, and low-quality reads. Genome assembly was performed using SPAdes 3.15.4 with k-mer sizes 55, 65, 75, and 95. Assembly quality was assessed using Quast 2.0 for statistical metrics and Busco 5.6.1 for completeness evaluation. The Genome Epidemiology Center analyzed assembled *E. coli* sequences to determine multi-locus sequence types (MLST), serotypes, plasmid replicons, virulence genes, and phylogenetic relationships. MLST 2.0 (Achtman scheme) identified sequence types, and cgMLSTFinder (https://bitbucket.org/genomicepidemiology/cgmlstfinder) was used to obtain core genome multilocus sequence typing (cgMLST) and allele profiles. A minimum spanning tree (MST) was constructed using GrapeTree (https://enterobase.readthedocs.io/en/latest/grapetree/grapetree-about.html). while SerotypeFinder 2.0 (90% identity, 60% coverage) and PlasmidFinder 2.1 (90% identity, 60% length) characterized serotypes and plasmid replicons, respectively. Virulence genes were annotated using Abricate 0.5 (VFDB database, default parameters). Twenty-two *E. coli* isolates were obtained in the present study (BioProject No. PRJNA1124013). Additionally, four non-pathogenic *E. coli* reference isolates—*E. coli* Nissle 1917 (NZ_CP007799.1), ATCC 25,922 (NZ_CP009072.1), EcAZ01 (OX_341604.1), and MG1655 (NZ_CP025268.1)—and four pathogenic *E. coli* isolates—AMSHJX01 (CP030939), AMSHJX02 (CP031105), AMSHJX03 (CP058355), and AMSHJX04 (CP058308)—originating from captive giant pandas in a different facility were retrieved from GenBank. Whole-genome SNPs were identified using Parsnp 2.0.3, and a maximum-likelihood phylogenetic tree was constructed with RAxML 8.2.13 (bootstrap = 1000). Antimicrobial resistance genes were predicted from SPAdes assemblies using ResFinder 4.1 and CARD. ANIb analysis was performed using pyani on 22 *E. coli* genomes [9]. Pairwise ANIb data were clustered and visualized via heatmap, with all software using default settings. Antimicrobial susceptibility testing followed CLSI guidelines using the Kirby-Bauer method [10]. Based on whole-genome analysis, eight types of antibiotics representing four categories were selected: fluoroquinolones, tetracyclines, aminoglycosides, and cephalosporins, considering both resistance profiles and veterinary clinical usage.

**Fig. 1:**
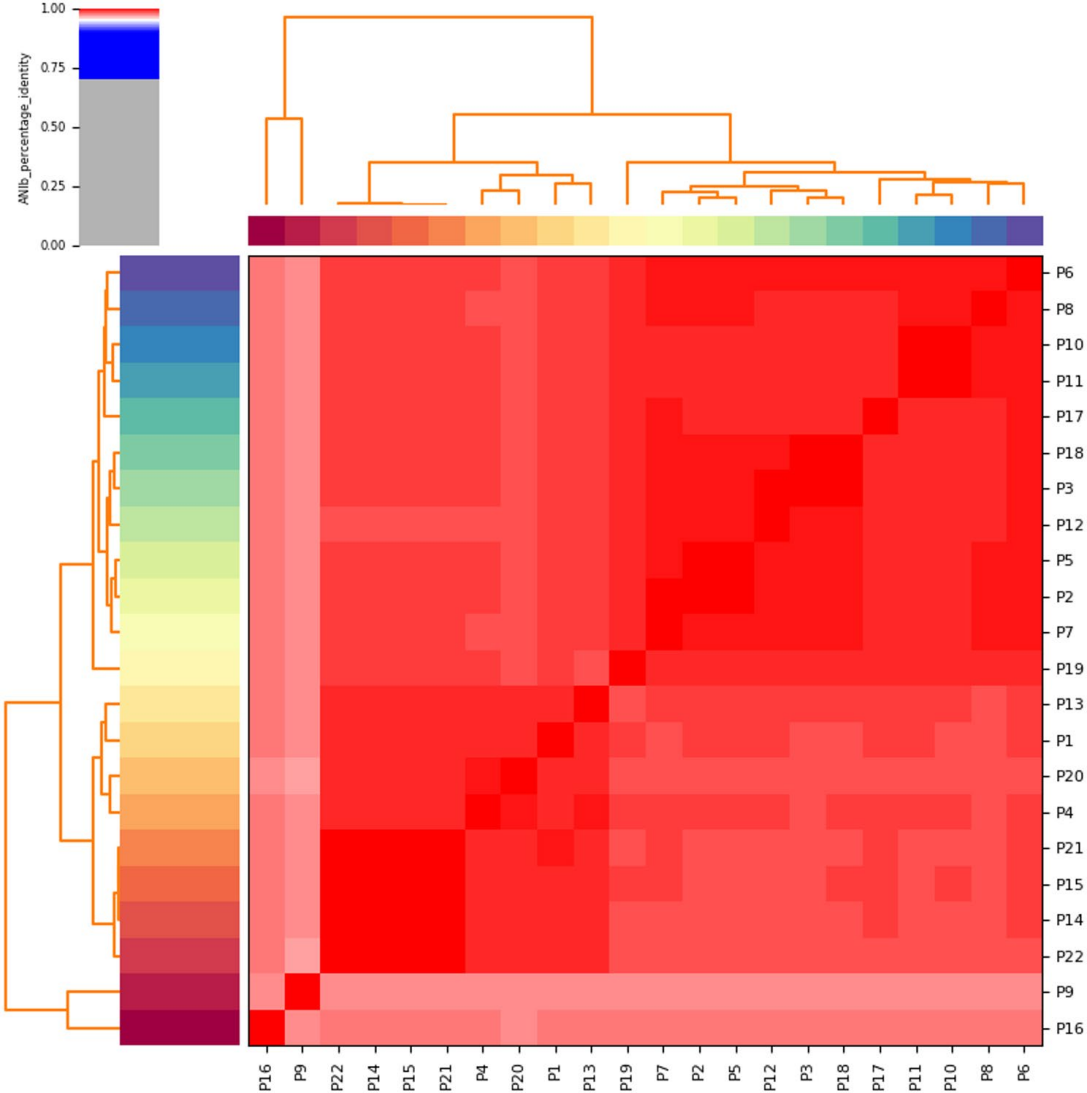
Heatmap representing the degree of similarity of the 22 *E. coli* genomes studied: The heatmap was derived from the average nucleotide identity (ANI) matrix based on BLAST(ANIb) approach. Color scheme varies from high similarity (red) to low similarity (grey) of the genomes analyzed (please see color range patterns and the corresponding similarity values highlighted in the bar legend of the figure

ANIb values (> 95%) among all 22 genomes confirmed their genetic relatedness and classification as the same *E. coli* species, supported by heatmap visualization (Fig. 1, Additional file 2). The genomes of the 22 captive giant panda-derived *E. coli* isolates were sequenced, with sizes of 4.61–4.92 Mbp, GC contents of 50.58–50.88%, and 4276–4649 coding sequences. BUSCO completion was 100% (see Additional file 3), confirming high-quality assemblies suitable for genome analysis.

**Fig. 2:**
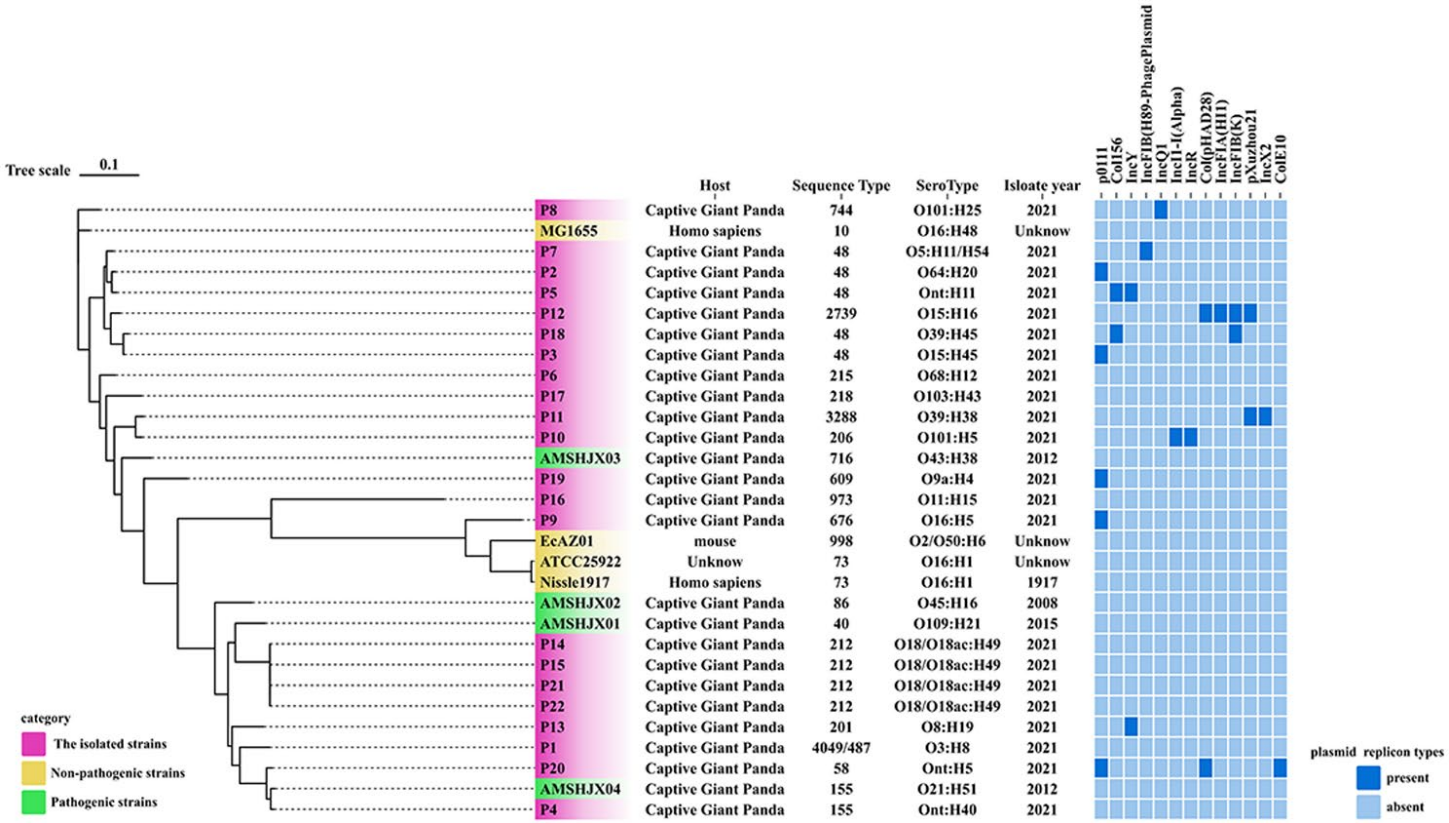
The evolutionary relationship of core genome-SNPs in the 22 *E. coli* isolates from captive giant pandas: The phylogenetic tree was constructed using the core SNPs identified from 22 *E. coli* genome sequences and *E. coli* reference genomes downloaded from NCBI. The analysis was performed using CSI Phylogeny 1.4 (https://cge.food.dtu.dk/services/CSIPhylogeny/) with the Maximum Likelihood method and a default bootstrap replication value of 1000. The *E. coli* phylogeny indicates (from left to right) host, sequence type (ST), serotype, isolation year and plasmid replicon types for each isolate

MLST analysis of 22 isolates identified 15 sequence types, with ST48 (5 isolates) and ST212 (4 isolates) being the most prevalent (Fig. 2), cgMLST analysis enhances strain typing resolution, enabling further differentiation of isolates within the same ST type. ST48 isolates are classified into multiple cgSTs (see Additional file 5), distributed across different evolutionary branches (Fig. 5), reflecting their genetic diversity. Plasmid replicon typing revealed p0111-type (22.7%) as the most common, alongside 11 other replicon types, including Col156, IncY, IncFIB(H89-PhagePlasmid), IncQ1, etc. (Fig. 2). Among the 22 *E. coli* isolates, 20 distinct serotypes were identified. Analysis revealed that the most prevalent serotype was O18/O18ac: H49, accounting for 18.18%. The remaining isolates displayed a diverse range of serotypes (Fig. 2). The 30 *E. coli* isolates were analyzed using core genome SNPs to build a phylogenetic tree, with isolates of similar sequence types and serotypes clustering together (Fig. 2). Although P9 clustered with non-pathogenic isolates (Nissle1917, EcAZ01, and ATCC25922) and P4 grouped with the pathogenic *E. coli* AMSHJX04, their actual pathogenicity needs to be determined through virulence gene prediction analysis combined with experimental validation. We detected a total of 88 virulence genes in the 22 *E. coli* isolates. To examine the variation in virulence gene profiles among these isolates, we constructed a presence/absence matrix showing the distribution of these virulence genes (Fig. 4). The matrix indicated that most isolates shared similar virulence gene profiles. The virulence genes present in our *E. coli* isolates mainly include Enterotoxins (*entA*, *fepA*), iron acquisition systems (*yagZ/Y*), and fimbriae-associated genes (*fimA/H*). These genes contribute to bacterial adhesion, immune evasion, and iron uptake.

**Fig. 3:**
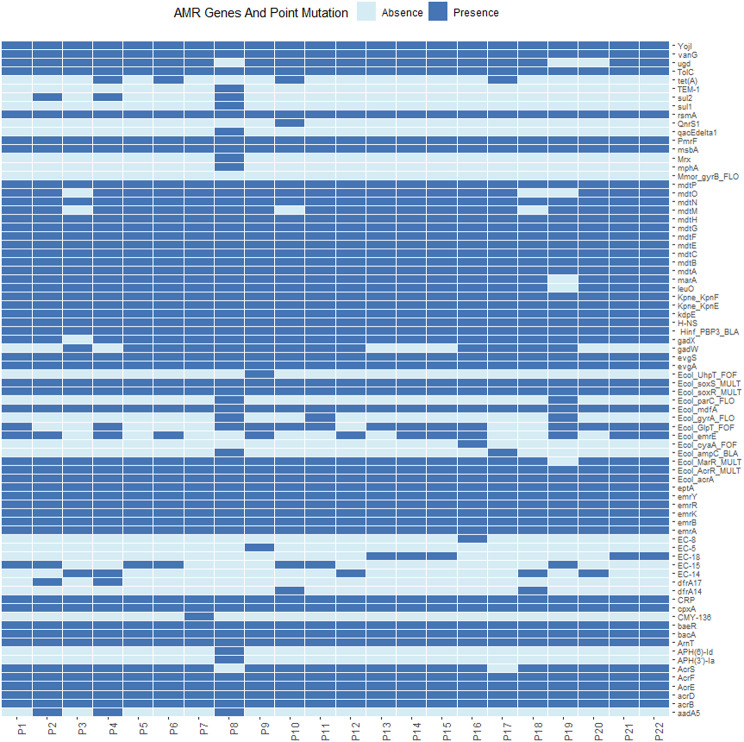
Profiles of AMR genes and point mutation for each isolate in this study

**Fig. 4:**
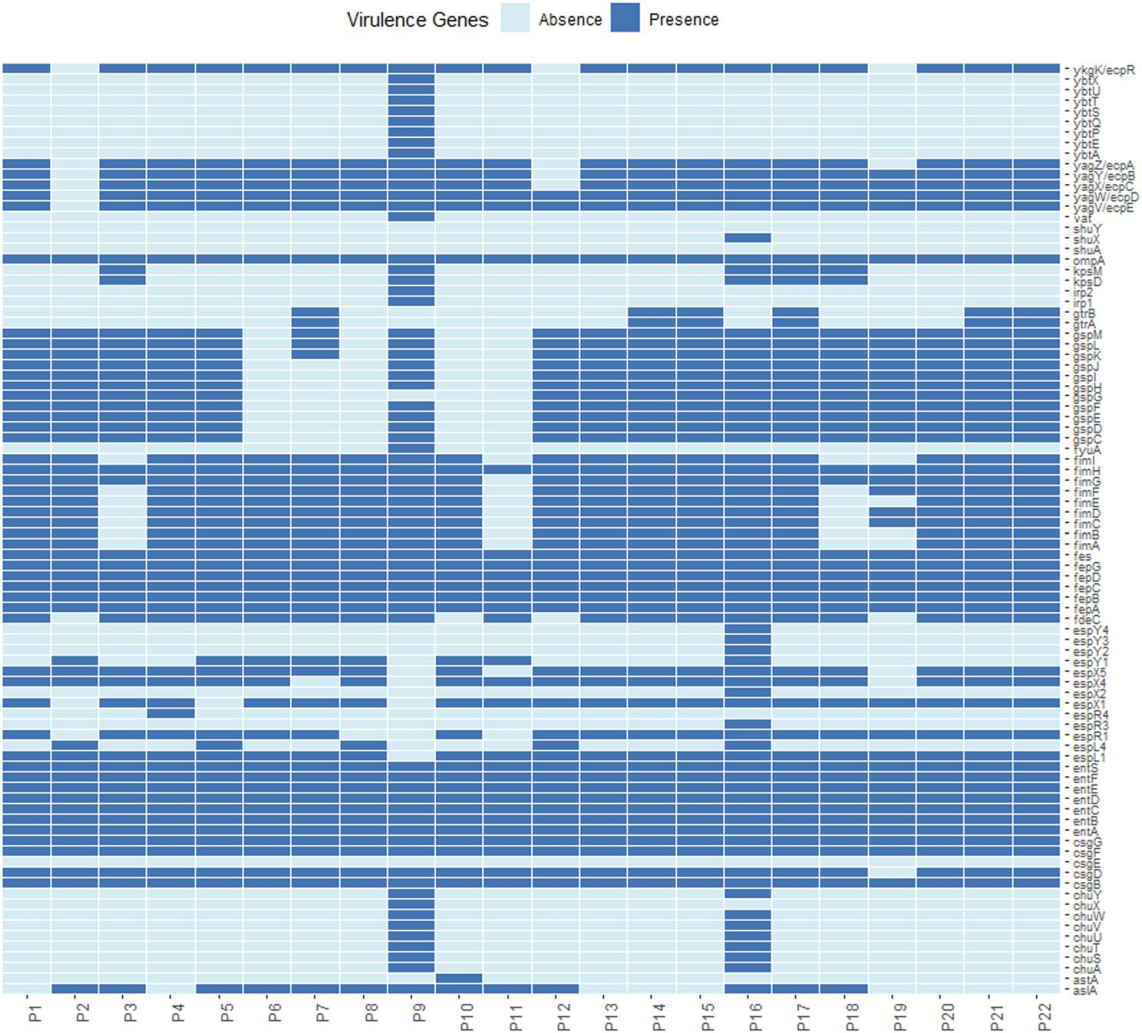
Profiles of Virulence Genes for each isolate in this study

**Fig. 5:**
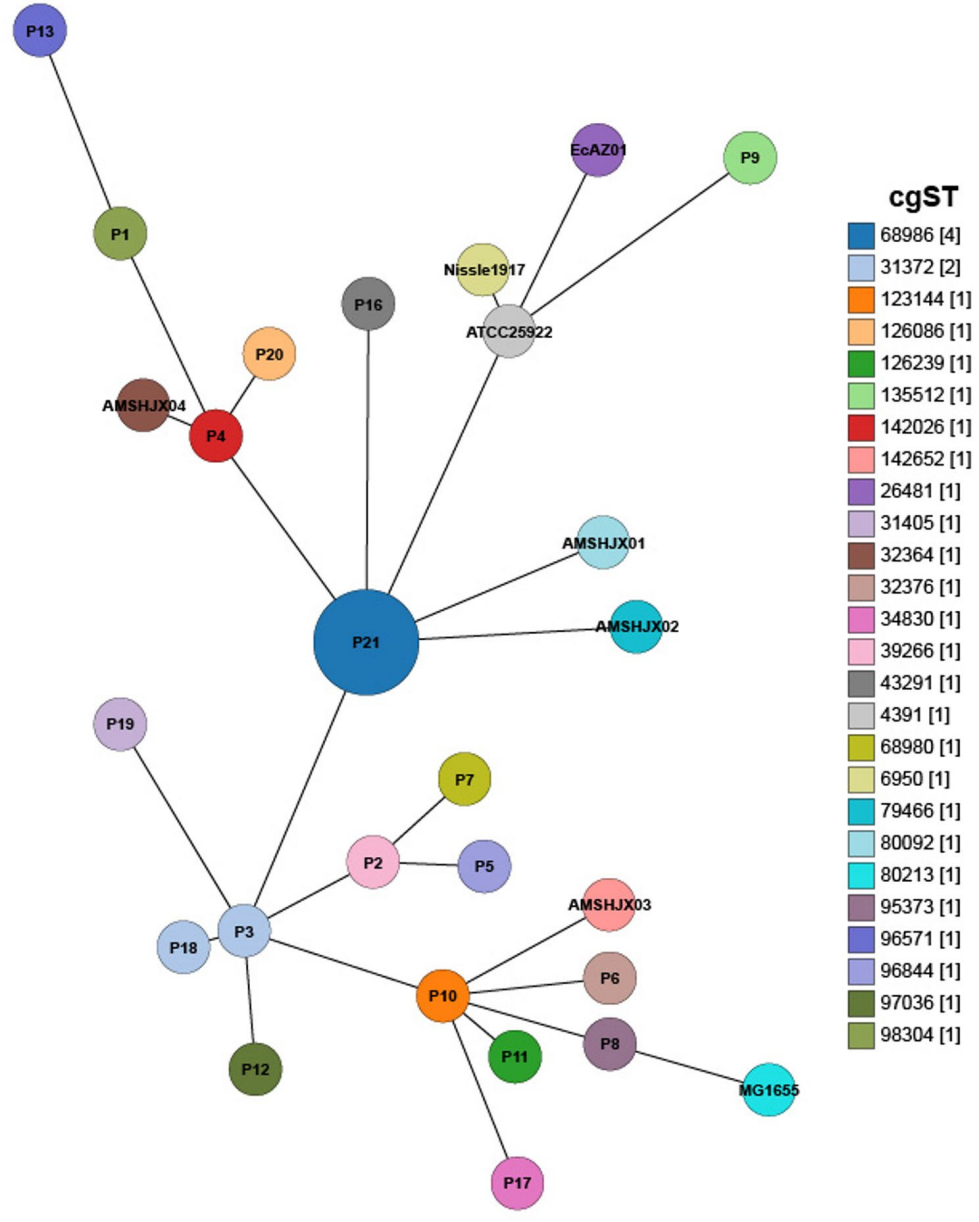
Minimum spanning tree of cgMLST of 30 *E. coli* isolates

Seventy-eight resistance genes and point mutations were identified in *E. coli* genomes, with fluoroquinolone resistance genes (*acrB*,* acrD*,* AcrE*,* AcrF*,* emrA*,* emrB*,* H-NS*, and *rsmA*) and tetracycline resistance genes (*emrK*,* emrY*,* evgA*, and *evgS*) being the most prevalent (Fig. 3). Antibiotic susceptibility testing showed all strains were sensitive to gentamicin, while only P14 was resistant to amikacin. The highest resistance rate was to ciprofloxacin (13/22; 59.1%), followed by norfloxacin (4/22; 18.2%) and tetracycline (3/22; 13.6%). Detailed resistance data is in Additional File 4.

*E. coli*, a well-studied intestinal bacterium, is ideal for investigating environmental impacts on gut microbiota and antibiotic resistance [11], especially in captive animals where antibiotic use may drive the selection of resistant isolates [12].

A total of 78 antibiotic resistance genes and point mutations were identified in *E. coli* isolates from 22 giant pandas, with minimal variation in gene numbers across isolates (Fig. 3), suggesting similar selective pressures in the captive environment influenced resistance gene retention. Especially, Fluoroquinolone resistance genes (e.g., *acrB*, *acrD*, *AcrE*, *AcrF*) were highly prevalent, linked to RND efflux pumps [13], likely due to ciprofloxacin’s common use in veterinary practice [14]. This correlates with a 59.1% ciprofloxacin resistance rate, suggesting the use of this antibiotic may drive resistance spread in captive giant pandas, significantly more intensively than other antibiotics (Additional file 4).

*E. coli* pathogenicity is driven by virulence genes that encode essential pathogenic factors [15]. Comparison with a virulence database identified 88 genes in the 22 *E. coli* isolates, with *fimH*, *fepA*, and *entA* being highly prevalent (Fig. 4). These virulence genes are linked to key pathogenic mechanisms in *E. coli*, including adhesion, invasion, and iron uptake [16]. For example, *fimH* encodes a fimbrial adhesion protein, likely enhancing host cell adhesion [17]. while *fepA* is involved in iron uptake and outer membrane formation [18, 19]. Their high prevalence suggests roles in bacterial survival and nutrient competition. Additionally, *entA*, associated with enterotoxin production, indicates potential toxin-mediated pathogenicity [20].

Analysis of O-antigen serotypes in 22 *E. coli* isolates revealed considerable diversity, with 19 classified into multiple serotypes and 3 untypeable (Fig. 2). O18 showed the highest detection rate and is associated with extraintestinal pathogenic *E. coli* (ExPEC), known for causing severe infections like meningitis and sepsis [21]. The O18 serotype exhibits high pathogenic potential across multiple hosts [22], likely due to shared core virulence factors that enhance cross-species transmission.

Isolates with identical MLST types, such as ST48 (P2, P3, P5, P7) and ST212 (P14, P15, P21, P22), underwent cgMLST to resolve their genetic relationships at a higher resolution. While ST212 isolates formed a single cgST, indicating a conserved genetic background, ST48 isolates were divided into multiple cgSTs, revealing significant genetic diversity within this ST type. These results demonstrate the utility of cgMLST in providing finer-scale resolution of isolate relationships compared to traditional MLST. P9 clusters with non-pathogenic strains (Nissle1917, EcAZ01, ATCC25922), suggesting it may also be non-pathogenic. In contrast, P4 is grouped with AMSHJX04 and shares the same MLST, ST155. Given that AMSHJX04 is a pathogenic *E. coli* strain isolated from a giant panda [23], this suggests that P4 may possess pathogenicity; however, further experimental validation is required.

Whole genome analysis of the 22 *E. coli* isolates revealed widespread resistance and virulence genes, including a high prevalence of ciprofloxacin resistance genes. While direct data on antibiotic usage in the facility are lacking, these findings may indicate selective pressure from prior antimicrobial exposure. The high prevalence of the O18 serotype, a hallmark of extraintestinal pathogenic *E. coli* (ExPEC), along with key virulence genes (e.g., *fimH*), hints at a potential risk for cross-host transmission, underscoring the need for vigilant health monitoring in giant pandas. These findings highlight the need for improved bacterial resistance monitoring in captive giant pandas to optimize antibiotic use and curb resistance gene spread.

## Electronic supplementary material

Below is the link to the electronic supplementary material.

Supplementary Material 1: Supplementary Table S1: Twenty-two isolated *E. coli* isolates with the basic information

Supplementary Material 2: Supplementary Table S2: ANI scores between the 22 *E. coli* isolates

Supplementary Material 3: Supplementary Table S3: General genome features of 22 *E. coli* isolates

Supplementary Material 4: Supplementary Table S4: Details of antimicrobial susceptibility test results for 22 *E. coli* isolates

Supplementary Material 5: Supplementary Table S5: cgMLST typing results of 30 *E. coli* isolates

## Data Availability

Raw sequencing reads were submitted to the National Center for Biotechnology Information (NCBI) under BioProjects No. PRJNA1124013.

## Declarations

### Prior publication

Data have not been published previously.

## Abbreviations

E. coli: Escherichia coli
MDR: Multidrug resistance
WGS: Whole-genome sequencing
MLST: Multi-Locus Sequence Typing
cgMLST: Core genome Multi-Locus Sequence Typing
ExPEC: Extraintestinal pathogenic Escherichia coli
MST: Minimum spanning tree
